# Photodynamic therapy of cancer‐associated infections

**DOI:** 10.1111/php.14038

**Published:** 2024-12-31

**Authors:** Giulia Kassab, Juan Chen, Gang Zheng

**Affiliations:** ^1^ Princess Margaret Cancer Centre University Health Network Toronto Ontario Canada; ^2^ Department of Medical Biophysics University of Toronto Toronto Ontario Canada

**Keywords:** bacteria, cancer, infections, photodynamic therapy, photosensitizers, virus

## Abstract

Pathogens can be involved in tumor initiation, promotion, and progression through different mechanisms, and their treatment can prevent new cancer cases, improve outcomes, and revert poor‐prognostic phenotypes. Photodynamic therapy (PDT) successfully treats different types of cancers and infections and, therefore, has a unique potential to address their combination. However, we believe this potential has been underutilized, and few researchers have investigated the impacts of PDT of both infection‐related and cancer‐related outcomes at once. This review presents the main agents behind cancer‐associated infections (CAIs), the PDT protocols that have been tested on them, and their key findings. Additionally, we discuss the key aspects of PDT that make it ideal for CAI treatment, and what knowledge gaps need to be filled in order to make it successful.

## Graphical abstract

Pathogens can be involved in tumor initiation, promotion and progression through different mechanisms, and their treatment can prevent new cancer cases, improve outcomes, and revert poor‐prognostic phenotypes. Photodynamic therapy (PDT) successfully treats different types of cancers and infections, and, therefore, has a unique potential to address their combination. This review presents the main agents behind cancer‐associated infections (CAIs), the PDT protocols that have been tested on them, and their key findings. Additionally, we discuss the key aspects of PDT that make it ideal for CAI treatment, and what knowledge gaps need to be filled in order to make it successful.

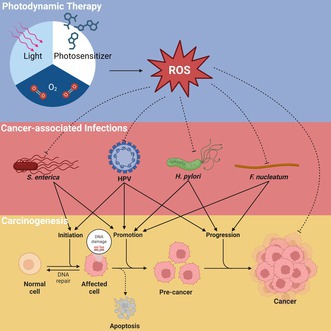

**Abbreviations**
5‐ALA: 5‐aminolevulinic acid
AlS_2_Pc: aluminum di‐sulfonated phthalocyanine
AlS_4_Pc: aluminum tetra‐sulfonated phthalocyanine
AlSPc: aluminum sulfonated phthalocyanines
AvrA: effector protein of *S. enterica*, named due to its similarity to the Avr (avirulence) family
CagA: cytotoxin‐associated antigen A
CAIs: cancer‐associated infections
CDT: cytolethal distending toxin
CdtB: subunit B of the cytolethal distending toxin
ETBF: enterotoxigenic *Bacteroides fragilis*
FadA: *Fusobacterium* adhesin A
Fap2: *Fusobacterium* apoptosis protein 2
HBV: hepatitis B virus
HCV: hepatitis C virus
HIV: human immunodeficiency virus
HMME: hematoporphyrin monomethyl ether
HPV: human papillomavirus
HTLV‐1: human T‐cell leukemia virus type 1
IARC: International Agency for Research on Cancer
KS: Kaposi sarcoma
MAL: methyl‐aminolevulinate
MB: methylene blue
PB031: a pyropheophorbide‐a derivative
PDT: photodynamic therapy
PDZ: photodithazine
pks: polyketide synthetase island
PP(Ala)2Arg2: a peptide‐conjugate porphyrin derivative
PpIX: protoporphyrin IX
ROS: reactive oxygen species
TBO: toluidine blue
THPTS: 3,3′’,3″”,3″”’‐(7,8,17,18‐tetrahydro‐21H,23H‐porphyrine‐5,10,15,20‐tetrayl) tetrakis[1‐ methyl‐pyridinium] tetratosylate
VacA: vacuolating cytotoxin auto transporter

## INTRODUCTION

It is estimated that, in 2018, 2.2 million new cases of cancer were attributable to infections.^1^ More specifically, these cancers were confirmed to have occurred after infection with at least one of 11 pathogens listed by the International Agency for Research on Cancer (IARC) as group 1 carcinogens, for which the causality has been confirmed with sufficient evidence in humans.^1^, ^2^ These include the gram‐negative bacterium *Helicobacter pylori*, several viruses, and a few parasites (see Table 1). However, not included in this estimation, are cases related to pathogens classified as group 2 carcinogens, for which the causality is deemed probable (group 2A) or possible (group 2B) by the IARC, but has not been confirmed (including *Plasmodium falciparum* and the Merkel cell polyomavirus).^2^ Additionally, there is accumulating scientific evidence that several other pathogens play a role in the occurrence, progression and severity of cancer (see selected in Table 2), and even specific strains of commensal and pathogenic bacteria that might do the same.^41^, ^42^, ^43^, ^44^, ^45^, ^46^, ^47^

**TABLE 1:** Studies of anti‐infectious photodynamic therapy in group 1 carcinogen infections.^*^

			PDT parameters						
Pathogen	Study type	PS	DLI	*λ*	*I*	*D*	Infection‐related outcome	Cancer‐related outcome	Source
Epstein‐Barr virus	*In vitro*	Zn (II)‐benzochlorin analog 2 μM	24 h	682 nm	0.8 mW/cm^2^	Up to 1 J/cm^2^	Not assessed	Up to 80% reduction in cell viability, cytokine modulation	^3^
	*In vitro*	HMME 16 μM	3 h	630 nm	10 mW/cm^2^	Up to 14.4 J/cm^2^	Not assessed	>90% reduction in cell viability, but EBV‐negative cells were more susceptible	^4^
HBV	*Ex vivo*	Riboflavin, up to 100 μM	None	Ultraviolet lamps	6 mW/cm^2^	3.6 J/cm^2^	Up to 70% viral DNA destruction	Not assessed	^5^
	*Ex vivo*	MB 1 μM	1 h	660 nm	272 mW/cm^2^	up to 979 J/cm^2^	Partial reduction of PCR signal	Not assessed	^6^
HCV	*Ex vivo*	MB 1 μM	1 h	660 nm	272 mW/cm^2^	up to 979 J/cm^2^	Partial reduction of PCR signal	Not assessed	^6^
	*Ex vivo*	MB 1 μM	1 h	fluorescent tubes	up to 50,000 lux	up to 50,000 lux‐hours	> 2 log reduction in PCR signal	Not assessed	^7^
	*Ex vivo*	MB 1 μM	None	660 nm	20 mW/cm^2^	up to 576 J/cm^2^	Complete loss of infectivity and >2 log reduction in PCR signal from circulating solution	Not assessed	^8^
HIV	*Ex vivo*	MB 1 μM	1 h	660 nm	272 mW/cm^2^	up to 979 J/cm^2^	Partial loss of PCR signal	Not assessed	^6^
	*Ex vivo*	MB 1 μM	1 h	Fluorescent tubes	up to 50,000 lux	up to 50,000 lux‐hours	>2 log reduction in PCR signal	Not assessed	^7^
	*In vitro*	Verteporfin 695 nM	1 h	690 nm	40–45 mW/cm^2^	up to 8.1 J/cm^2^	Up to > 3 log reduction in viral titers	Not assessed	^9^
	*Ex vivo*	Verteporfin up to 695 nM	1 h	690 nm	40–45 mW/cm^2^	15 J/cm^2^	Up to > 2 log reduction in viral titers	Not assessed	^9^
HPV	Clinical	Topical 5‐ALA gel (118 mg/g) as adjuvant therapy	3–4 h	635 nm	100–150 mW/cm^2^	100 J/cm^2^	Significantly fewer infected lesions after 6 months when compared to standard therapy alone (from 80% to 24%)	Not assessed	^10^
	Clinical	Topical 5‐ALA gel (118 mg/g) as adjuvant therapy	3–4 h	635 nm	80 mW/cm^2^	144 J/cm^2^	Three sessions led to significant increase in cure rates and negative conversions of pre‐cancerous lesions at 3 and 6 months	Not assessed	^11^
	Clinical	Topical MAL cream (200 mg/g)	3 h	630 nm	80 mW/cm^2^	100 J/cm^2^	10 sessions achieved similar efficacy but lower recurrence rate than the gold‐standard treatment for pre‐cancerous lesions	Not assessed	^12^
	Clinical	Topical 5‐ALA thermogel (236 mg/g)	4 h	635 nm	100 mW/cm^2^	100 J/cm^2^	3 sessions led to 56.1% infection clearance in patients without visible lesions	Not assessed	^13^
	*In vitro*	Verteporfin (0.5 μM) or N‐Aspartyl chlorine e6 (20 μM)	1 h	690 or 660 nm	2 mW/cm^2^	up to 200 mJ/cm^2^	Not assessed	HPV‐infected cancer cells were more resistant to PDT than non‐infected cells	^14^
	*In vitro*	Curcumin (free or nanoemulsion, up to 120 μM)	3–24 h	447 nm	209 mW/cm^2^	50 J/cm^2^	Not assessed	PDT effect was similar in infected and non‐infected cancer cells, and non‐infected healthy cells	^15^
*H. pylori*	*In vitro*	Ruthenium complex‐loaded micrometric glass beads (3.4 mg/g)	None	465 nm	14.1 mW/cm^2^	up to 25.4 J/cm^2^	Up to > 7 log reduction in CFU/mL	Not assessed	^16^
	*In vitro* and *ex vivo*	Benzylidene cyclopentanone photosensitizers (up to 10 μM)	30 min	532 nm	40 mW/cm^2^	24 J/cm^2^	Significant reduction of CFU in vitro (> 7 log) and ex vivo (< 1 log), with some toxicity to human gastric cells	Not assessed	^17^
	*In vitro*	MB (up to 625 μM)	30 min	Endoscopic white light	940 lux	up to 157 lux‐hours	Complete (>8 log) reduction of CFU/mL	Not assessed	^18^
	*In vitro*	AlSPc (up to 2.5 mM)	4 h	675 nm	Not informed	1.5 or 5.5 J/cm^2^	Complete bacterial inactivation (initial inoculum not informed)	Not assessed	^19^
	*In vitro*	Endogenous porphyrins	None	White light or 405 nm	100 mW/cm^2^	up to 30 J/cm^2^	Up to 6 log reduction of survival fraction	Not assessed	^20^
HTLV‐1	*In vitro*	Hypericin (up to 396 nM)	16 h	520–750 nm	6.27 mW/cm^2^	11.28 J/cm^2^	Significant suppression of viral gene expression (<1 log) but no reduction in cell‐to‐cell transmission	Significant reduction in viability and clone efficiency of cancerous and transformed T‐cells	^21^
	*In vitro* and *ex vivo*	5‐ALA acid (1 mM)	24 h	400–700 nm	Not informed	9.6 J/cm^2^	Not assessed	Selective killing of leukemic cells over healthy cells	^22^
	*Ex vivo*	5‐ALA (up to 1 mM)	4 h	630 nm	20.4 mW/cm^2^	80.8 J/cm^2^	Not assessed	Eradication of acute ATL tumor cells with mild to no toxicity to normal cells or other malignancies	^23^
Kaposi sarcoma Herpesvirus	Clinical	Intravenous indocyanine green (2–4 mg/kg weight)	1–30 min	805 nm	0.5–5 W/cm^2^	100 J/cm^2^	Not assessed	Complete remission 4 weeks after treatment	^24^
	Clinical	Topical MB and TBO (20 mg/g)	30 s	600–750 nm	100 mW/cm^2^	18 J/cm^2^	Not assessed	Clinical remission achieved after around 5 sessions	^25^

^*^ According to the IARC.^2^ No applications of PDT were found for *Clonorchis sinensis*, *Opisthorchis viverrini*, or *Schistosoma haematobium*.

**TABLE 2:** Selected studies of anti‐infectious photodynamic therapy in other cancer‐associated infections.

			PDT parameters						
Pathogen	Study type	PS	DLI	*λ*	*I*	*D*	Infection‐related outcome	Cancer‐related outcome	Source
*Campylobacter jejuni*	*In vitro*	Curcumin (up to 2.71 mM) or chlorophyllin (up to 1.38 mM)	None	430 nm	0.15 mW/cm^2^	45 mJ/cm^2^	No effect	Not assessed	^26^
*Clostridioides difficile*	*In vitro*	MB, talaporfin, chlorin e_6_, AlSPc, and pyro‐pheophorbide derivatives (up to 100 μM)	5 min	665 nm	18 mW/cm^2^	0.24 J/cm^2^	Talaporfin, chlorin e6, S4 and PB031 reduced bacterial counts by >4 log even in anaerobic conditions	PB031 was toxic to HT‐29 colon cancer cells, but not talaporfin nor chlorin e_6_	^27^
*Fusobacterium nucleatum*	Clinical	Topical MB (31.2 mM)	3 min	670 nm	75 mW	4.5 J	Treatment failed to reduce *F. nucleatum* burden in periodontal treatment	Not assessed	^28^
	*In vitro*	TBO (10 μM)	30 min	830 nm	0.1 W	3 J	5 log reduction in bacterial viability	Not assessed	^29^
	*In vitro*	TBO (330 μM)	None	650 nm	60 mW/cm^2^	18 J/cm^2^	2 log reduction in bacterial viability	Not assessed	^30^
	*In vitro*	PDZ (up to 102 μM)	10 min	660 nm	71.7 mW/cm^2^	50 J/cm^2^	In biofilm, treatment resulted in 1 log reduction of viable bacterial concentration	Not assessed	^31^
	*In vitro*	Curcumin (270 μM), PpIX (178 μM), resazurin (436 μM) or riboflavin (266 μM)	None	405 nm	84.5 mW/cm^2^	25.3 J/cm^2^	PpIX aPDT significantly reduced the optical density of 24‐hour cultures; curcumin was also effective but had dark toxicity	Not assessed	^32^
	*In vitro*	MB (up to 20 μM)	None	665 nm	484 mW/cm^2^	14.53 J/cm^2^	Complete bacterial inactivation (>5 log) with 20 μM	Not assessed	^33^
Merkel cell polyomavirus (group 2A^*^)	Clinical	Intratumoral PP(Ala)_2_Arg_2_ (918 μM)	24 h	632.8 nm	60 mW	50 J/cm^2^	Not assessed	Chemo‐ and radiotherapy resistant lesions responded successfully to the PDT treatment (1 patient)	^34^
*Morganella morganii*	Clinical veterinary	MB (0.1 mg/g)	5 min	660 nm	3.5 W/cm^2^	280 J/cm^2^	*M. morganii* was present in one of the snake subjects and gone after treatment	Not assessed	^35^
	*In vitro*	THPTS (up to 200 μM)	20 min	420 or 760 nm	13 or 18 mW/cm^2^	Up to 93.6 or 116.6 J/cm^2^	Limited efficacy (< 1 log reduction)	Not assessed	^36^
*Plasmodium falciparum* (group 2A^*^)	*In vitro*	5‐ALA (up to 2 mM)	8 h	White light	570 mW/cm^2^	Up to 1026 J/cm²	Significant reduction of parasitemia (from about 3% to 0%); no sign of erythrocyte toxicity	Not assessed	^37^
	*In vitro*	Pheophorbide‐a containing magnetic nanoparticles (up to 0.65 μM)	24 h	667 nm	6.58 mW/cm²	Up to 23.69 J/cm²	Parasitemia was reduced from 6‐8% to 0% after treatment	Not assessed	^38^
	*In vitro*	Merocyanine 540 (26.3 μM)	None	White light (> 470 nm)	2.6 mW/cm²	14 J/cm²	Parasitemia reduction from 10% to 0% at low hematocrit, and to about 3% at high hematocrit	Not assessed	^39^
	*In vitro*	5‐ALA (50 μM)	4–6 h	635 nm	150 mW/cm²	9 J/cm²	Parasitemia reduction from 20–40% to 0–3% after multiple PDT sessions	Not assessed	^40^
*Peptostreptococcus anaerobius*	*In vitro*	MB (up to 20 μM)	none	665 nm	484 mW/cm^2^	14.53 J/cm^2^	>4 log bacterial inactivation with 20 μM	Not assessed	^33^

^*^ According to the IARC (2). No applications of PDT were found for group 2B carcinogen *Schistosoma japonicum*.

**FIGURE 1:**
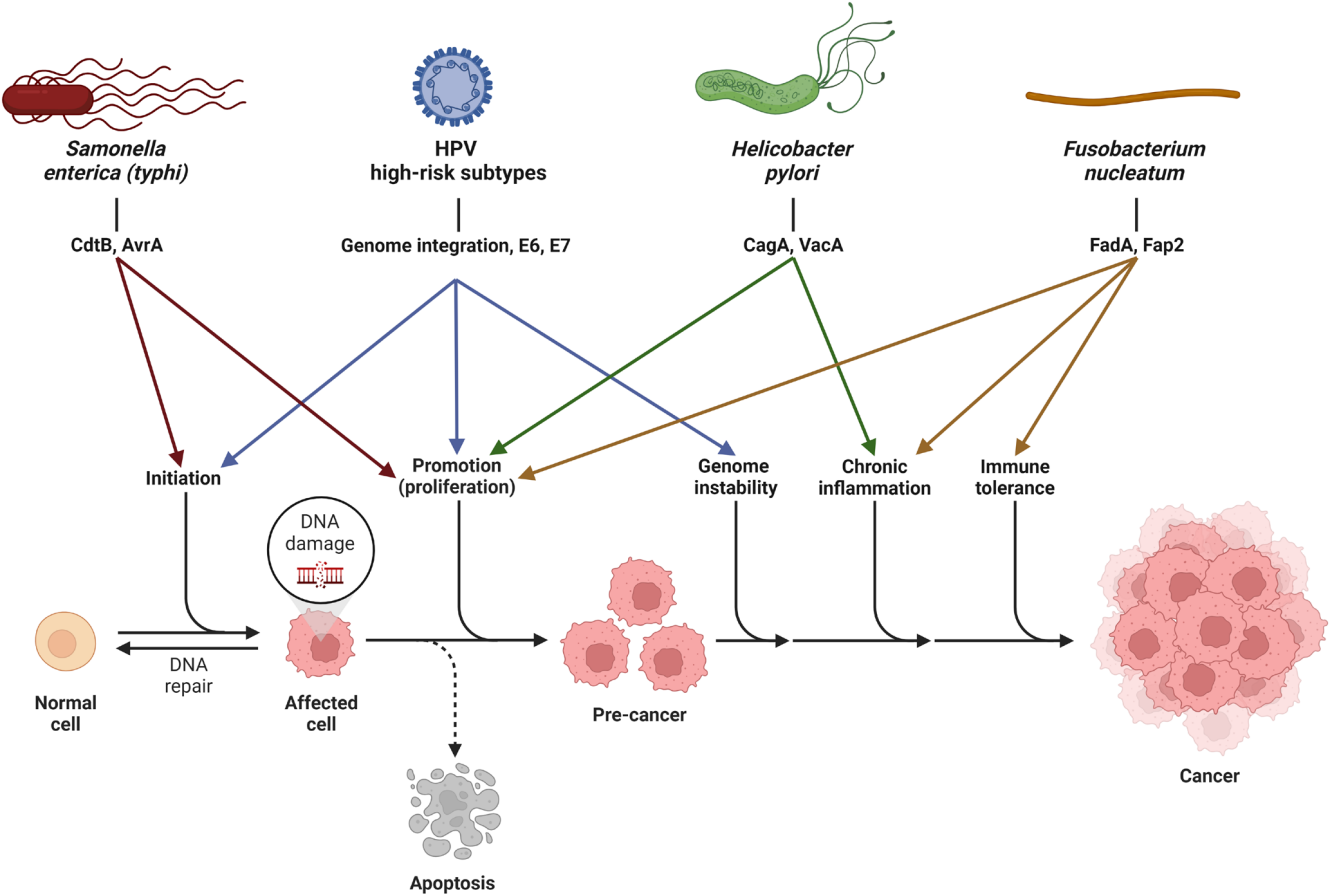
Examples of mechanisms through which pathogens induce and enhance carcinogenesis. Made using biorender.com.

Pathogens can be involved in tumor initiation, promotion, and progression through different mechanisms (Figure 1). For example, the human papillomavirus (HPV) and other oncoviruses insert their genome into the DNA of the host cells, causing mutations that may lead to tumor initiation. In high‐risk subtypes like HPV16 and HPV18, the expression of oncoproteins E6 and E7 impairs growth regulatory pathways, creating the opportunity for mutated cells to thrive. Successful screening and treatment of pre‐cancerous HPV lesions are known to significantly reduce the incidence of cervical cancers.^48^, ^49^, ^50^ *Salmonella enterica* subsp. *typhi* is proposed to be carcinogenic through a similar mechanism: the cytolethal distending toxin subunit B (CtdB) of the typhoid toxin damages DNA, while the effector protein AvrA activates Wnt‐β‐catenin signaling, disabling growth regulation and leading to tumor promotion.^41^ However, not all CAIs are direct DNA mutagens. *H. pylori* induces cell proliferation through the cytotoxin‐associated antigen A (CagA, which also acts on Wnt‐β‐catenin signaling), then promotes chronic inflammation through the vacuolating cytotoxin auto transporter (VacA).^41^, ^51^, ^52^ In *Fusobacterium nucleatum*, the *Fusobacterium* adhesin A (FadA) activates Wnt‐β‐catenin signaling and promotes chronic inflammation, while the *Fusobacterium* apoptosis protein 2 (Fap2) inhibits the cytotoxic function of natural killer cells, leading to immune tolerance.^41^, ^53^, ^54^ In a mouse model, antibiotic treatment of *F. nucleatum*‐infected colon cancer increased overall survival and elicited an immune response effective against rechallenge with both infected and uninfected cancer cells.^46^ The use of antibiotics as adjuvants in cancer treatment has shown some success in clinical trials.^46^, ^55^ However, antibiotic treatment success is limited by the occurrence of resistance and by localized delivery challenges.^45^, ^56^

Photodynamic therapy (PDT) combines the use of a photosensitizer, light and molecular oxygen to generate cytotoxic oxidative damage that is not specific to a single molecular target. Yet, it can be designed to selectively target cancers or pathogens, and has been safely implemented into clinical practice for the treatment of several cancers.^57^ Despite not being as widely researched and adopted as an anti‐infectious treatment, PDT has many advantages over traditional antibiotics.^58^ Thus, it presents itself as a promising treatment for cancer‐associated infections (CAIs), both in the prevention of cancer, and as adjuvant for its treatment.

In this review, we present the main pathogens in CAIs, the PDT protocols that have been tested on them, and their key findings. Additionally, we discuss the key aspects of PDT that make it ideal for CAI treatment, and what knowledge gaps need to be filled in order to make it successful.

## PHOTODYNAMIC THERAPY OF CANCER‐ASSOCIATED PATHOGENS

### Confirmed cancer‐causing pathogens (group 1 human carcinogens)

*H. pylori* is the only bacterial pathogen classified as group 1 on the IARC list.^2^ It is, however, the leading cause of infection‐attributed cancers, corresponding to over a third of all new cases in 2018.^1^ PDT for *H. pylori* has been previously investigated *in vitro* and *ex vivo*, and showed significant success (Table 1). In a study from 1990, Bedwell *et al*. showed complete inactivation of six patient‐derived *H. pylori* strains using a mixture of aluminum sulfonated phthalocyanines (AlSPc) and 675 nm.^19^ In the same study, the rat colonic mucosa was used as a proxy for the stomach lining, and the PDT treatment led to local necrosis that healed safely. However, a follow‐up study using the isolated aluminum di‐sulfonated phthalocyanine (AlS_2_Pc) revealed that its accumulation and effect in the blood vessels could be harmful to the stomach, and concluded that 5‐aminolevulic acid (5‐ALA), a porphyrin prodrug, would be a more adequate photosensitizer for the gastric mucosa.^59^ Later, Hamblin *et al*. found that *H. pylori* accumulates endogenous porphyrins, even more, do that *Propionibacterium acnes*, and so white or blue‐light treatment alone was sufficient to cause >5 log killing *in vitro*.^20^ Since then, other photosensitizers and treatment therapies have been investigated.^16^, ^17^, ^18^ However, none of these studies addressed the impact of PDT on *H. pylori*'s carcinogenesis or infected cancer cells, nor have we been able to find any studies that do.

Most of the other group 1 carcinogenic pathogens are viruses. On one hand, viral particles have no ability to repair oxidative damage and thus are easily destroyed by PDT.^60^ On the other hand, viral DNA incorporated into host cells can be incredibly difficult to target. One clever strategy makes use of the virus‐induced cell proliferation (see Figure 1): 5‐ALA and methyl‐aminolevulinate (MAL) are prodrugs, converted into protoporphyrin IX (PpIX) and other photosensitizing porphyrins inside the cell. In faster‐proliferating cells, this conversion occurs more rapidly, creating a level of selectivity against the surrounding healthy tissue that allows for both imaging and PDT.^61^, ^62^ Several small‐scale clinical trials using 5‐ALA and MAL have shown encouraging results in the PDT treatment of pre‐cancerous HPV infection, with evidence of high efficacy, low recurrence, and immune modulation.^10^, ^11^, ^12^, ^13^, ^63^ 5‐ALA has also been tested against malignant T‐cells transformed by the human T cell leukemia virus type 1 (HTLV‐1), which accumulate PpIX 10 to 100‐fold more than healthy cells, leading to a significant difference in cytotoxicity *in vitro* and *ex vivo*.^22^, ^23^ Porphyrin derivatives have also been investigated, but were not as selective as the prodrugs. Li *et al*. used hematoporphyrin monomethyl ether (HMME) as a PDT agent against the Epstein–Barr virus, but found that it was more effective in non‐infected than infected human cells.^4^ Similarly, Kessel *et al*. found that the benzoporphyrin derivative Verteporfin was more effective in HPV‐negative than HPV‐positive cell lines.^14^

Methylene Blue (MB) has also often been used in the photodynamic inactivation of viral infections, as there is evidence that it selectively binds to viral particles.^64^ MB has shown encouraging results in the decontamination of plasma and other blood products containing the human immunodeficiency virus (HIV) type 1, the hepatitis B virus (HBV), and the hepatitis C virus (HCV).^6^, ^7^ In a case study of an HIV‐related Kaposi sarcoma (KS) patient, several chemotherapy‐resistant lesions were treated with PDT using a combination of MB and toluidine blue (TBO) and cure was achieved after about five sessions, leading to a completely negative biopsies.^25^ In a similar case study, indocyanine green was given to three KS patients, and lesions selectively accumulated the photosensitizer, showing fluorescence in the infrared region.^24^ Four weeks after a single PDT treatment, complete remission was confirmed through histopathology. Other photosensitizers used for viral CAIs are listed in Table 1.

Finally, the flukes *Schistosoma haematobium*, *Opisthorchis viverrini,* and *Clonorchis sinensis* are also classified by IARC as confirmed carcinogens. As we have found no publications investigating PDT for these pathogens, they were not included in the table. Nonetheless, there is some evidence of successful PDT in another trematode: in 2017, de Melo *et al*. showed that toluidine‐based PDT targets the tegument and excretory system of *Schistosoma mansoni*, leading to a near‐complete inhibition of parasite activity 4 days after treatment in vitro.^65^

### Other cancer‐associated pathogens

The IARC lists *Plasmodium falciparum* and Merkel Cell Polyomavirus as group 2A (probable) carcinogens, and *Schistosoma japonicum* as a group 2B (possible) carcinogen.^2^ As listed in Table 2, a single patient with Merkel cell carcinoma was included in a case study after being refractory to chemo‐ and radiotherapy and received PDT with a peptide‐conjugated porphyrin (PP(Ala)_2_Arg_2_). After 40 days, even the largest tumors receded.^34^ 5‐ALA, merocyanine 540, and a pheophorbide‐a carrying nanoparticle all showed to be effective against *P. falciparum* and protective toward new erythrocyte infection *in vitro*.^37^, ^38^, ^39^, ^40^ As for *S. japonicum*, similarly to the group 1 flukes, no PDT protocols were found.

There is increasing scientific evidence for the role of several other pathogens in carcinogenesis, which have yet to be listed by the IARC. Table 2 also includes some of these agents and PDT protocols that have been tested on them. *F. nucleatum* is probably the pathogen with the most evidence of involvement in carcinogenesis outside the IARC list: besides the previously mentioned role of the proteins Fap2 and FadA in oncogenesis, *F. nucleatum* seems to also be involved in cancer metastasis, recurrence, and poor prognosis.^66^, ^67^ In a randomized clinical trial proposing to address periodontitis using MB, no difference was seen in the extent of *F. nucleatum* colonization of the mouth after one PDT session.^28^ *In vitro*, however, MB‐based PDT completely inactivates this pathogen.^33^ TBO, the chlorin Photodithazine (PDZ), and PpIX also seem to be effective *in vitro*.^29^, ^31^, ^32^

*Campylobacter jejuni* produces the cytolethal distending toxin (CDT), which promotes DNA‐strand breaks and was shown to potentiate carcinogenesis in a mouse model.^41^ Urrutia *et al*. attempted PDT against *C. jejuni in vitro* using chlorophyllin and curcumin, but the proposed protocols were unsuccessful.^26^ *Clostridioides difficile* seems to be able to activate Wnt‐β‐catenin signaling, and it is frequently present in pancreatic ductal adenocarcinoma.^41^, ^45^ De Sordi *et al*. screened 13 different photosensitizers against *C. difficile* and found that while most of them were active in normal *in vitro* conditions, only talaporfin, chlorin e_6_, the aluminum tetra‐sulfonated phthalocyanine (AlS_4_Pc) and the modified pyropheophorbide‐a PB031 were active in anaerobic conditions, which more accurately represent the *in vivo* conditions.^27^ The same study showed that all four photosensitizers partially inactivated *C. difficile* spores and that PB031 was active against colon cancer cells. *Morganella morganii* has shown preclinical evidence of being a CAI, which is backed by its expression of indolimines, small molecules that promote cell cycle arrest, and DNA damage.^41^ In a veterinary case study, a snake with *M. morganii*‐positive stomatitis was treated with MB‐based PDT once a week for 3 months. After the treatment period, the lesion had not fully healed, but no longer contained *M. morganii*.^35^ The porphyrin derivative 3,3′,3″,3″’‐(7,8,17,18‐tetrahydro‐21H,23H‐porphyrine‐5,10,15,20‐tetrayl) tetrakis[1‐ methyl‐pyridinium] tetratosylate (THPTS) was tested against *M. morganii in vitro*, but only showed limited efficacy.^36^ *Peptostreptococcus anaerobius* also potentiated tumorigenesis in a mouse model, and research suggests that it induces enhanced cell proliferation, NF‐κB activation, infiltration of myeloid‐derived suppressor cells, and increased intracellular reactive oxygen species (ROS).^41^ Rai *et al*. showed that MB is a successful photosensitizer against *P. anaerobius*, with a light dose‐dependent and concentration‐dependent inactivation *in vitro*.^33^

### Strain‐specific cancer‐associated pathogens

Some examples of literature‐supported CAIs are strain‐specific. In *Escherichia coli*, strains that carry the *polyketide synthetase* (*pks*) island express colibactin, a toxin that induces interstrand cross‐links and double‐strand DNA breaks in colonic epithelial cells.^41^, ^68^ *E. coli* has been an extensive target for PDT research, and several photosensitizers have been shown successful in its killing.^69^, ^70^, ^71^, ^72^ However, we found no specific study of PDT in *E. coli pks* ^+^ or of its effect in colibactin. *S. enterica* subsp. *typhi*, as previously mentioned, is proposed to induce carcinogenesis due to the expression of the typhoid toxin.^41^ PDT protocols using eosin, Rose Bengal and 5‐ALA have been tested in *S. enterica* subsp. *typhimurium*, a non‐typhoid strain.^73^, ^74^ While it is likely that PDT would be also effective against the *typhi* serovar, more studies are necessary, particularly in regard to the effect of the typhoid toxin and its effect on cancer. *Bacteroides fragilis* is known for promoting vitamin D‐dependent tumor resistance, but enterotoxigenic *B. fragilis* (ETBF) is implicated in colon cancer tumorigenesis.^75^, ^76^ Nitzan *et al*. tested the efficacy of deuteroporphyrin‐based PDT against 42 clinical isolates of *B. fragilis*, out of which only one was not successfully inhibited, but the biological differences between strains were not investigated.^77^ Then, Cassidy *et al*. achieved great photodynamic inactivation using 5‐ALA and an oxygen‐releasing compound on the *B. fragilis* strain NCTC 9343.^78^ Yet, this is a non‐toxigenic strain.^79^

## POTENTIAL BENEFITS OF PDT FOR CAIs

Tables 1 and 2 show that, despite there having been several studies of PDT for CAI‐related pathogens, they have rarely investigated infection‐related and cancer‐related outcomes simultaneously. In fact, the study of hypericin‐based PDT on HTLV‐1 and adult T‐cell leukemia by Xu et al. seems to be the only one that aimed to target and quantify the effects on both pathogen and cancer cells.^21^ De Sordi *et al*. investigated the effect of *C. difficile*‐targeted PDT on colon cancer cells as a proxy for host‐cell toxicity, and their goal was to find a PDT‐protocol that was effective on the bacteria without harming the mammalian cells.^27^ Even the several HPV clinical studies that highlight the importance of infection treatment on cancer prevention show no follow‐up on the cancer rates of treated patients. Moreover, very little consideration has been paid to strain‐specific CAIs in PDT research. Yet, PDT combines unique characteristics that make it ideal for concurrently addressing the infection and cancer concerns of CAIs.

First, PDT can evidently address several types of pathogens including viruses, gram‐negative and gram‐positive bacteria, protozoa, and animal parasites, as presented in this review, as well as fungi. But it is spatially selective, since the ROS generation only occurs in the area exposed to light. This is protective to the healthy microbiome, as opposed to traditional antibiotic and antiparasitic treatment (which can lead to adverse effects, dysbiosis, and even be pro‐cancerous).^80^ Particularly in the case of cancer patients, antibiotic treatment is considered a double‐edged sword: beneficial when targeting CAIs, but harmful when non‐specifically affecting the microbiome.^46^, ^80^, ^81^ In addition to the spatial selectivity, PDT agents can be designed and modified to target specific pathogens.^82^, ^83^

Secondly, anti‐infectious PDT is often performed at milder conditions than cancer PDT, using lower photosensitizer concentrations, shorter drug‐light intervals, lower irradiances, and smaller light doses. Thus, it would be helpful in circumstances where cancer‐targeting PDT might be too aggressive (as seen, for example, by Guidolin *et al*. on a colon cancer model^84^). And more importantly, these milder PDT protocols should still be able to activate an immune response. Inflammation and immune cell recruitment are known to be important factors in the successful PDT of both cancers and infections.^85^ This has been explored in PDT‐based cancer vaccines, in which patient‐specific neoantigens are created by *ex vivo* treatment of their tumor cells.^86^ Combining anti‐cancer PDT with the exposure to viral epitopes (in a non‐viral cancer model) primed the humoral immune response and induced CD8^+^ T‐cell activation, leading to increased efficacy and prevention of recurrence *in vivo*.^87^ Therefore, it would be logical to expect that the anti‐infectious PDT treatment of CAIs would promote a cancer‐preventing and cancer‐treating immune response. However, the underlying mechanisms of anti‐infectious PDT immune activation have been understudied, as well as their impacts on cancer.

## CONCLUSION

Anti‐infectious photodynamic therapy protocols have been investigated for many confirmed and proposed cancer‐associated pathogens, ranging from in vitro to clinical studies, and with varying degrees of success. However, the impact of these treatments on cancer prevention, treatment, and prognostics has often been overlooked. Similarly, studies of cancer‐focused PDT have often failed to acknowledge the role of cancer‐causing infections. We believe that since anti‐infectious PDT can target a broad spectrum of pathogens while sparing the healthy microbiome, and can induce an immune‐modulating response directed at both microbes and cancer cells, it can be harnessed to treat CAIs and simultaneously achieve positive infection and cancer outcomes.

Yet, much research is still necessary to confirm and best harness the properties of PDT in CAIs. Firstly, CAI pathogens and strains should be considered in experimental design; then, in vitro studies should simultaneously assess infection and cancer‐related outcomes (including infection load, cell viability, clonogenic assays, selective photosensitizer uptake, and the effect of PDT on specific carcinogenesis pathways), and finally, clinical studies should follow‐up over time and assess cancer incidence in PDT‐treated and non‐treated patients. This way, the true potential of PDT for this application will be uncovered.
